# Supplementary material for: Improved haplotype resolution of highly duplicated MHC genes in a long-read genome assembly using MiSeq amplicons
Source: PeerJ. 2023 Jul 12;11:e15480. doi: 10.7717/peerj.15480 (PMC10349553; doi:10.7717/peerj.15480)
Supplement: Supplemental Information 1 — The number of unique amplicon alleles of expected length and post-trimming length are reported only for the focal individual. The number of MHC-I amplicon alleles that had deletions leading to a shift in open-reading frame in exon 3 are stated within brackets for each primer pair (PP) combination (in total seven MHC-I alleles had such deletions when considering sequences from both MHC-I PP).The number of MHC-IIB amplicon alleles that contained stop-codons are stated within brackets for each primer pair (PP) combination (in total ten MHC-IIB alleles contained premature stop-codons when considering sequences from both MHC-IIB PP). Fewer unique amplicon alleles were found with two MHC-IIB PPs (PP2 and PP3) and data from both PPs were discarded before the trimming and the mapping steps. [file peerj-11-15480-s001.docx]

| Target (locus) | Primer pairs (PP) | Forward primer sequence (5'–3') | Reverse primer sequence (5'–3') | Expected length (bp) | Number of unique sequences (of expected length) | Post-trimming length (bp) | Number of unique sequences post-trimming |
| --- | --- | --- | --- | --- | --- | --- | --- |
| MHC-I | HNalla/HN46 | TCCCCACAGGTCTCCACAC | ATCCCAAATTCCCACCCACCTT | 263 | 16 (5) | 263 | 16 (5) |
| MHC-I | HNalla-1/R3Ex3b | CCCCACAGGTCTCCACAC | TTGYGCTCYAGCTCCTTC | 246 | 22 (6) | 246 | 22 (6) |
| MHC-IIB | PP1:  MHC2Aca_ex2F_us28-us9/MHC2Aca_ex2R_ds27-ds10 | CCCCCTGACCTGTGTCCTGC | RGGGGACACGCTCTGCCC | 287 | 86 (10) | 270 | 84 (9) |
| MHC-IIB | PP2:  MHC2Aca_ex2F_us26-us8/  MHC2Aca_ex2R_ds94-ds75 | CCCTGACCTGTGTCCTGCA | GAGGGCTCTGGGGTGATTCC | 351 | 74 |  |  |
| MHC-IIB | PP3:  MHC2Aca_ex2F_us26-us8/  MHC2Aca_ex2R_ds26-ds9 | CCCTGACCTGTGTCCTGCA | GGGGACACGCTCTGCCCY | 285 | 65 |  |  |
| MHC-IIB | PP5:  MHC2_ex2F_us17-ex7b/ MHC2_ex2R_ds17-ex7 | GTGTCCTGCACACYCAGGGGTGTT | CTCTGCCCYGCRCTCACCTCGGCG | 256 | 79 (10) | 256 | 79 (10) |
