# Supplementary material for: Improved haplotype resolution of highly duplicated MHC genes in a long-read genome assembly using MiSeq amplicons
Source: PeerJ. 2023 Jul 12;11:e15480. doi: 10.7717/peerj.15480 (PMC10349553; doi:10.7717/peerj.15480)
Supplement: Supplemental Information 2 — Number of MHC-I and MHC-IIB amplicon allele sequences that match to annotated MHC alleles in genome assemblies (GRW Falcon-2017 and Purge Haplotigs) of the focal individual. The number of amplicons alleles being mapped has been assessed using the standard Geneious RNA mapper (Custom Sensitivity, allowing 0% to 4% mismatches per read). [file peerj-11-15480-s002.docx]

|  |  | Mismatches allowed | | | | |
| --- | --- | --- | --- | --- | --- | --- |
|  | Genome version: | 0% | 1% (2 bp) | 2% (5 bp) | 3% (7–8 bp) | 4% (10 bp) |
| MHC-I  (29 alleles) | GRW Falcon-2017 | 16 | 19 | 20 | 21 | 21 |
|  | Purge Haplotigs | 13 | 13 | 14 | 15 | 15 |
| MHC-IIB  (95 alleles) | GRW Falcon-2017 | 54 | 77 | 78 | 78 | 78 |
|  | Purge Haplotigs | 48 | 71 | 74 | 75 | 75 |
