# Supplementary material for: Improved haplotype resolution of highly duplicated MHC genes in a long-read genome assembly using MiSeq amplicons
Source: PeerJ. 2023 Jul 12;11:e15480. doi: 10.7717/peerj.15480 (PMC10349553; doi:10.7717/peerj.15480)
Supplement: Supplemental Information 3 — All primary and associated MHC-I scaffolds (Aaru-UA scaffold) included in the GRW Falcon-2017 are presented. Scaffolds indicated with # were discarded after the post-assembly procedure was performed in the Purge Haplotigs assembly. Amplicon alleles were separated into three categories related to their inheritance in the focal individual: paternal alleles (P, blue), maternal alleles (M, yellow) and unresolved alleles (U, turquoise). Annotated alleles not detected with amplicon mapping are highlighted in grey. One amplicon allele was successfully amplified in the focal individual (both replicates) but not found in its parents and have the prefix “Seq”. The mapping procedure was performed in Geneious Prime® (Geneious RNA mapper). Non-functional genes are indicated with the symbol Ψ . [file peerj-11-15480-s003.docx]

|  |  | Mismatches allowed | | | |
| --- | --- | --- | --- | --- | --- |
| Aaru-UA scaffold | Acar-UA allele | 0% | 1% (2 bp) | 2% (5 bp) | 3% (7 bp) |
| 508 | 1 | U-9 | - | - | - |
|  | 2**Ψ** | P-11 | - | - | - |
|  | 3 | P-7 | - | - | - |
|  | 4 | P-7 | - | - | - |
|  | 5 | U-6 | - | - | - |
|  | 6 | P-10 | - | - | - |
|  | 7 | - | - | M-29 | P-13 |
|  | 8 | M-27 | - | - | - |
|  | 9 | U-6 | - | - | - |
|  | 10 | P-7 | - | - | - |
|  | 11 | P-19 | - | - | - |
| 18 | 1 | Seq73 | - | - | - |
|  | 2**Ψ** | U-1 | - | - | - |
|  | 3 | U-46 | - | - | - |
| 61 | 1 | U-43 | - | - | - |
|  | 2 | U-8 | - | - | - |
| 45 | 1 | - | - | - | - |
| 112 | 1**Ψ** | P-5 | - | - | - |
| # 91 | 1 | U-43 | - | - | - |
| # 4483 | 1 | P-33 | - | - | - |
| # 828 | 1**Ψ** | P-4 | - | - | - |
| # 6596 | 1**Ψ** | M-28 | P-17; U-35; P-42 | - | - |
| # 7324 | 1**Ψ** | M-28 | P-17; U-35; P-42 | - | - |
| # 4502 | 1 | - | - | - | - |
| # 2874 | 1**Ψ** | - | - | - | - |
