# Supplementary material for: Improved haplotype resolution of highly duplicated MHC genes in a long-read genome assembly using MiSeq amplicons
Source: PeerJ. 2023 Jul 12;11:e15480. doi: 10.7717/peerj.15480 (PMC10349553; doi:10.7717/peerj.15480)
Supplement: Supplemental Information 4 — All primary and associated MHC-IIB scaffolds (Aaru-DAB scaffold) included in the GRW Falcon-2017 are presented (including 96 and four annotated MHC-IIB alleles, respectively). Scaffolds indicated with # were discarded after the post-assembly procedure was performed in the Purge Haplotigs assembly. Amplicon alleles were separated into three categories related to their inheritance in the focal individual: paternal alleles (P, blue), maternal alleles (M, yellow) and unresolved alleles (U, turquoise). Additional amplicon alleles that were corresponding to multiple hits with higher mismatches are indicated with numbers (based on matrix of similarity). Annotated MHC alleles not detected or not assigned with amplicon mapping are highlighted in grey. The mapping procedure was performed in Geneious Prime® (Geneious RNA mapper). Non-functional genes are indicated with the symbol Ψ . [file peerj-11-15480-s004.docx]

|  |  | Mismatches allowed | | | |
| --- | --- | --- | --- | --- | --- |
| Aaru-DAB scaffold | Acar-DAB allele | 0% | 1% (2 bp) | 2% (5 bp) | 3% (8 bp) |
| 554 | 1 | M-64 | - | - | - |
|  | 2 | M-74 | - | - | - |
|  | 3 | M-79 | - | - | - |
|  | 4 | - | M-84 | - | - |
|  | 5 | M-100 | - | - | - |
|  | 6 | M-107 | - | - | - |
|  | 7 | M-3 | - | - | - |
|  | 8 | M-93 | - | 1 | - |
|  | 9 | M-67 | - | - | - |
| 357 | 1 | U-122 | - | - | - |
|  | 2 | P-134 | - | - | - |
|  | 3 | P-34 | - | - | - |
|  | 4 | - | - | - | - |
|  | 5**Ψ** | - | - | - | - |
|  | 6 | P-115 | - | - | - |
|  | 7 | P-130 |  | 1 | - |
|  | 8 | P-36 | - | - | - |
|  | 9 | P-53 | - | - | - |
| 120 | 1**Ψ** | U-132 | P-32; M-148; 3 | 4 | - |
|  | 2 | - | - | - | - |
|  | 3**Ψ** | U-132 | P-32; M-148; 3 | 4 | - |
|  | 4 | P-40 | M-27 | 2 | 1 |
|  | 5 | U-138 | 1 | 10 | - |
|  | 6 | - | 1 | 10 | 2 |
|  | 7 | U-91 | M-58 | 1 | - |
|  | 8 | M-7 (PP1) | - | 4 | 7 |
|  | 9 | - | 1 | 1 | 6 |
|  | 10 | U-50 | - | 2 | - |
|  | 11**Ψ** | U-6 (PP5) | 1 | - | - |
|  | 12 | M-52 | P-153; 1 | 7 | 1 |
|  | 13 | P-35 | M-9; 2 | 1 | 4 |
|  | 14 | M-111 | - | 2 | - |
|  | 15**Ψ** | M-1 (PP1) | - | 2 | - |
|  | 16 | M-52 | P-153; 1 | 7 | 1 |
|  | 17 | M-97 | 2 | - | 4 |
|  | 18 | M-101 | P-153; 1 | 8 | 1 |
|  | 19**Ψ** | U-6 (PP5) | 1 | - | - |
| 45 | 1**Ψ** | P-109 | P-131; 2 | 5 | 2 |
|  | 2 | P-17 | M-16; P-129 | 3 | - |
|  | 3 | P-151 | M-150; 1 | 2 | - |
|  | 4 | - | 8 | 3 | - |
|  | 5 | - | - | - | - |
|  | 6 | - | 2 | 1 | - |
|  | 7 | M-9 (PP1) | - | 3 | 8 |
|  | 8 | U-45 | M-89; 1 | - | - |
|  | 9 | U-117 | - | 8 | - |
| 301 | 1 | U-1 | P-31; 6 | M-140; 3 | - |
|  | 2 | - | M-5 (PP5) | 9 | - |
|  | 3**Ψ** | M-86 | P-142; U-120; 2 | 3 | 3 |
|  | 4 | P-11 | U-61; M-121; 1 | 1 | 3 |
|  | 5**Ψ** | P-104 | 3 | 4 | 3 |
|  | 6 | U-2 (PP1) | 7 | 1 | 2 |
| 18 | 1 | U-56 | - | - | - |
|  | 2**Ψ** | U-7 | - | - | - |
|  | 3 | U-20 | U-126; 1 | 6 | 3 |
|  | 4 | - | - | - | - |
| 168 | 1 | U-127 | 6 | 2 | - |
|  | 2 | U-102 | - | 4 | 3 |
|  | 3 | M-116 | - | 6 | 6 |
| 178 | 1 | P-112 | 6 | 2 | - |
|  | 2 | - | 8 | 4 | - |
| 104 | 1 | P-14 | M-89; 1 | - | - |
| 96 | 1 | U-38 | 4 | 5 | 2 |
| 505 | 1 | - | 7 | 2 | 2 |
| 3427 | 1 | P-39 | 3 | 6 | 2 |
| 6045 | 1 | U-71 | M-24 | 1 | - |
| # 91 | 1 | - | M-143 | 2 | 2 |
| # 721 | 1 | M-111 | - | - | - |
| # 1074 | 1**Ψ** | - | - | - | - |
|  | 2 | M-52 | P-153; 1 | 7 | 1 |
|  | 3 | U-61 | 2 | 4 | 2 |
| # 1508 | 1 | U-1 | P-31; 6 | M-140; 3 | - |
|  | 2 | U-1 | P-31; 6 | M-140; 3 | - |
| # 1773 | 1 | U-45 | M-89; 1 | - | - |
|  | 2 | M-121 | 1 | 5 | 2 |
|  | 3 | U-120 | M-5 (PP1); M-85; P-131; 4 | 1 | 2 |
| # 2695 | 1 | U-1 | P-31; 6 | M-140; 3 | - |
|  | 2 | - | - | - | - |
| # 3022 | 1 | U-1 | P-31; 6 | M-140; 3 | - |
|  | 2 | - | 8 | 2 | 2 |
| # 3118 | 1 | U-2 (PP1) | P-59; P-118; P-30; U-10; M-5 (PP5); 2 | 1 | 2 |
|  | 2 | U-120 | PP1-M-5; M-85; P-131; 4 | 1 | 2 |
|  | 3 | M-9 | 2 | - | - |
| # 3640 | 1 | U-38 | 4 | 5 | 2 |
| #4578 | 1 | - | 3 | 7 | - |
| # 5062 | 1 | M-111 | - | 2 | - |
| #5252 | 1 | - | 1 | 1 | 6 |
|  | 2**Ψ** | - | - | - | - |
| # 5399 | 1 | - | 8 | 2 | 2 |
|  | 2 | U-1 | P-31; 6 | M-140; 3 | - |
| # 5638 | 1 | U-1 | P-31; 6 | M-140; 3 | - |
| # 5980 | 1 | U-120 | M-5 (PP1); M-85; P-131; 4 | 1 | 2 |
| # 6215 | 1 | U-45 | M-89; 1 | - | - |
|  | 2 | - | P-11; 4 | 1 | 3 |
|  | 3**Ψ** | M-86 | P-142; 4 | 3 | - |
|  | 4 | U-2 (PP1) | P-59; P-118; P-30; U-10; PP5-M-5; 2 | 1 | 2 |
|  | 5 | U-1 | P-31; 6 | M-140; 3 | - |
| # 6600 | 1 | - | P-118; 7 | 1 | - |
| # 6738 | 1 | U-38 | 4 | 5 | 2 |
| # 7015 | 1 | - | U-1; 7 | 2 | 2 |
