# Supplementary material for: Improved haplotype resolution of highly duplicated MHC genes in a long-read genome assembly using MiSeq amplicons
Source: PeerJ. 2023 Jul 12;11:e15480. doi: 10.7717/peerj.15480 (PMC10349553; doi:10.7717/peerj.15480)
Supplement: Supplemental Information 5 — Mean amino-acid pairwise distances (below diagonal) and standard errors (standard error in blue, above diagonal) between annotated scaffolds including tandemly duplicated MHC-IIB gene copies in open reading frame (ORF) in the focal individual: Acar-DAB*120_2;4–10;12–14;16–18, Acar-DAB*301_1;2;4;6, Acar-DAB*357_1–4;6–9, Acar-DAB*45_2–9 and Acar-DAB*554_1–9. [file peerj-11-15480-s005.docx]

|  | Aaru-DAB*120 | Aaru-DAB*301 | Aaru-DAB*357 | Aaru-DAB*45 | Aaru-DAB*554 |
| --- | --- | --- | --- | --- | --- |
| Aaru-DAB*120 |  | 0.015 | 0.015 | 0.014 | 0.015 |
| Aaru-DAB*301 | 0.134 |  | 0.016 | 0.014 | 0.016 |
| Aaru-DAB*357 | 0.123 | 0.126 |  | 0.015 | 0.013 |
| Aaru-DAB*45 | 0.133 | 0.127 | 0.126 |  | 0.015 |
| Aaru-DAB*554 | 0.125 | 0.131 | 0.093 | 0.128 |  |
