# Supplementary material for: Improved haplotype resolution of highly duplicated MHC genes in a long-read genome assembly using MiSeq amplicons
Source: PeerJ. 2023 Jul 12;11:e15480. doi: 10.7717/peerj.15480 (PMC10349553; doi:10.7717/peerj.15480)
Supplement: Supplemental Information 6 — Out of 29 MHC-I amplicon alleles 16 were mapping to annotated alleles and out of 95 MHC-IIB amplicon alleles 58 were mapping. The upper panel represents amplicon alleles that mapped to the assembly and are separated into two categories: alleles mapping once (numbers in black, percentages in orange) and alleles mapping multiple times (numbers in blue, percentages in brown). Out of the 25 annotated MHC-I alleles, 21 were detected by amplicon alleles and out of the 100 annotated MHC-IIB alleles, 78 were detected by amplicon alleles.The lower panel describes the annotated MHC alleles in the GRW Falcon-2017 assembly, which were detected by amplicon alleles, and is separated into two categories: alleles detected uniquely (numbers in black, percentages in orange) and different alleles detected by the same amplicon allele (i.e., shared; numbers in blue, percentages in brown). [file peerj-11-15480-s006.pdf]

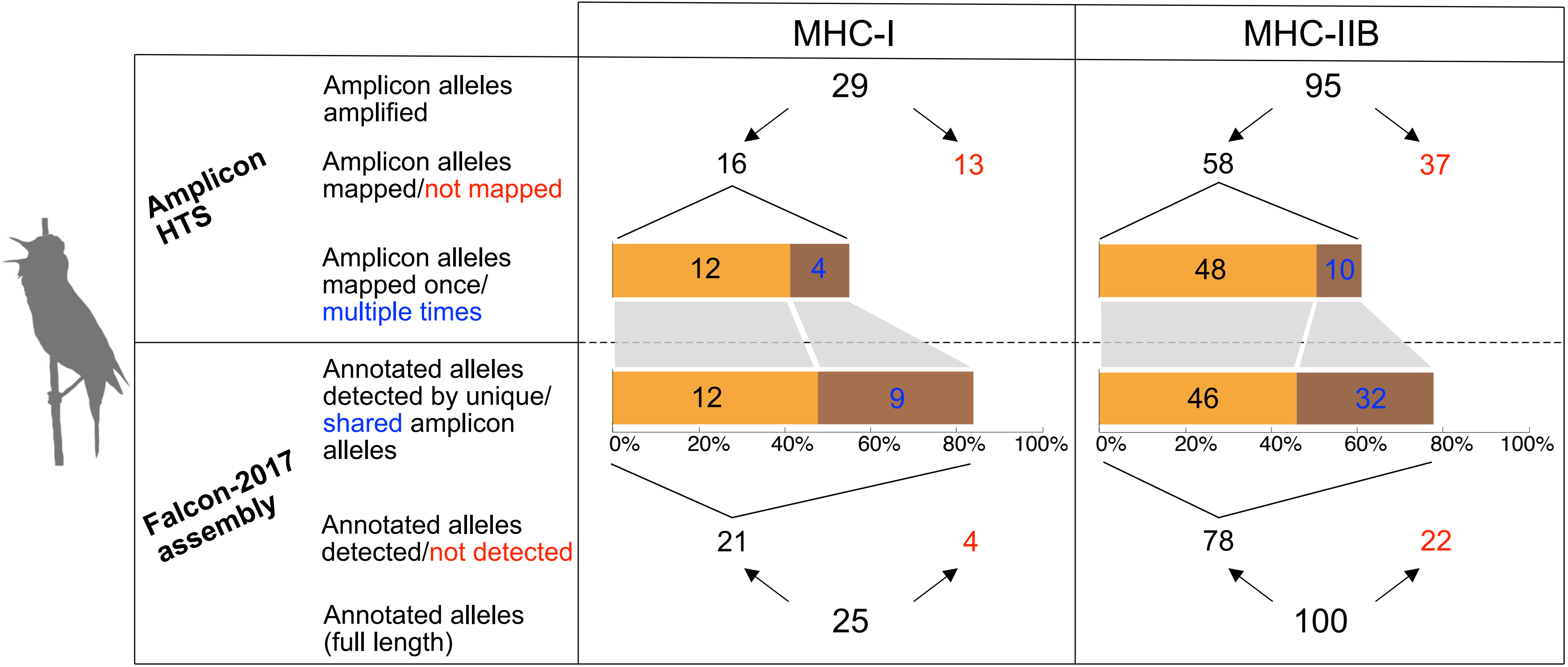
MHC-I
MHC-IIB
Amplicon HTS
Falcon-2017 assembly
Amplicon alleles amplified
Amplicon alleles mapped/not mapped
Amplicon alleles mapped once/ multiple times
Annotated alleles detected by unique/ shared amplicon alleles
Annotated alleles detected/not detected
Annotated alleles (full length)
29
16
13
12
4
12
9
21
4
25
95
58
37
48
10
46
32
78
22
100
0%
20%
40%
60%
80%
100%
