# Supplementary figures and images for: Improved haplotype resolution of highly duplicated MHC genes in a long-read genome assembly using MiSeq amplicons
Source: PeerJ. 2023 Jul 12;11:e15480. doi: 10.7717/peerj.15480 (PMC10349553; doi:10.7717/peerj.15480)

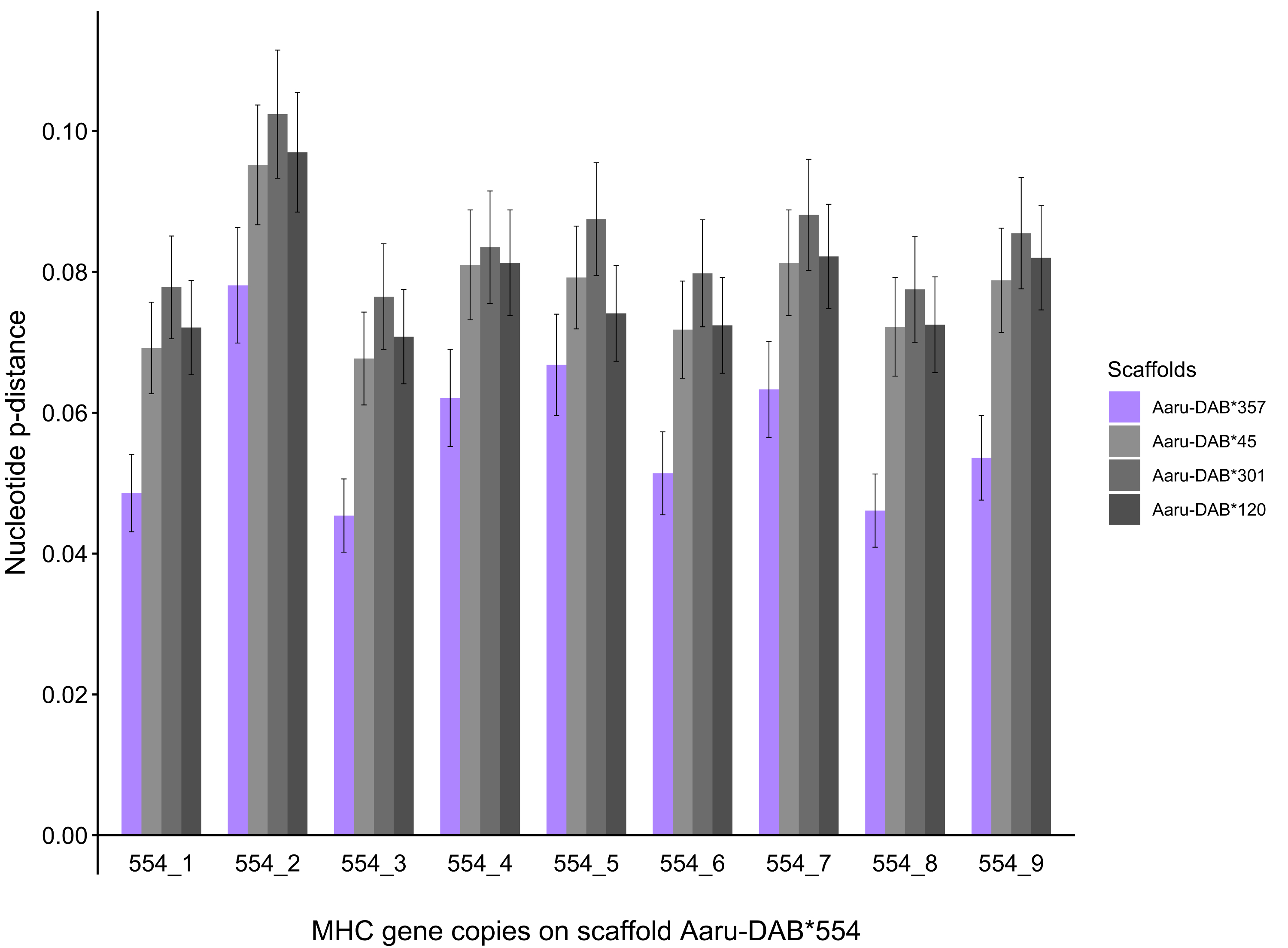

Supplement: Supplemental Information 7 — Mean nucleotide p-distances computed between each MHC-IIB gene copy from scaffold Aaru-DAB*554 (Acar-DAB*554_1–9) and all MHC-IIB gene copies at four scaffolds with tandemly duplicated genes in open reading frame (ORF) in the focal individual: Aaru-DAB*357 (purple), Aaru-DAB*45 (light grey), Aaru-DAB*301 (medium grey) and Aaru-DAB*120 (dark grey). [file peerj-11-15480-s007.jpg]
